# Is yoga more beneficial than exercise-based interventions for patients with chronic low back pain? A systematic review and meta-analysis

**DOI:** 10.3389/fmed.2026.1792208

**Published:** 2026-04-14

**Authors:** Tian Li, Quan Wen, Liping Zhang

**Affiliations:** 1 School of Rehabilitation, Gannan Medical University, Ganzhou, Jiangxi, China; 2 Communist Youth League Committee, Gannan Medical University, Ganzhou, Jiangxi, China; 3 College of Sports and Arts, JiangXi University of Science and Technology, Ganzhou, Jiangxi, China

**Keywords:** chronic low back pain, disability, emotional wellbeing, exercise therapy, meta-analysis, pain, physiotherapy, systematic review

## Abstract

**Background:**

Chronic low back pain (CLBP) is a prevalent condition associated with persistent pain, functional limitations, and psychological distress. Yoga is frequently recommended as an active, mind body intervention, yet it remains uncertain whether yoga provides added benefit compared with other exercise based programs.

**Objective:**

To compare the effects of yoga versus active exercise interventions on pain intensity, physical function or disability, and emotional wellbeing in adults with CLBP.

**Methods:**

A systematic review and meta-analysis was conducted in line with PRISMA 2020 and the Cochrane Handbook, and registered in PROSPERO. PubMed, Embase, Cochrane Library, Web of Science, and PEDro were searched from inception to November 23, 2025. Parallel-group randomized controlled trials enrolling adults with non-specific CLBP were included when they compared yoga with exercise based interventions and reported extractable continuous outcomes. Standardized mean differences (SMDs) with 95% confidence intervals (CIs) were pooled using fixed or random effects models based on heterogeneity. Risk of bias was assessed with the Cochrane tool.

**Results:**

Seven randomized controlled trials were included. For pain, yoga was not statistically superior to exercise comparators (SMD = –0.52, 95% CI –1.38–0.35; *I*^2^ = 95%). For physical function, yoga showed a significant improvement compared to exercise controls (SMD = –1.20, 95% CI –1.64 to –0.77; *I*^2^ = 31%); however, this specific finding is based on a limited evidence pool of only two studies. For disability, no clear between-group difference was observed (SMD = -0.19, 95% CI –1.11–0.73; *I*^2^ = 92%). For emotional outcomes, yoga improved emotional wellbeing compared with exercise controls (SMD = –0.71, 95% CI –1.26 to –0.16; *I*^2^ = 75%), and this effect became more consistent after removing one influential study (*I*^2^ = 0%).

**Conclusion:**

Based on the results of the current meta-analysis, yoga demonstrated statistically significant improvements in physical function and emotional wellbeing compared to active exercise interventions, while no consistent advantage was observed for pain or disability. These findings are specific to the limited evidence base and high heterogeneity identified in this study; therefore, while the data indicates positive trends, yoga’s comparative superiority should be viewed as a preliminary observation from this synthesis rather than a definitive clinical conclusion.

## Introduction

Persistent lower back discomfort (PLBD), typically characterized as lumbar region pain lasting for a minimum of 3 months, represents a widespread and debilitating musculoskeletal disorder that significantly diminishes overall wellbeing and physical performance (1). Beyond nociceptive symptoms, chronic low back pain (CLBP) is increasingly conceptualized through a biopsychosocial framework in which pain intensity, disability, emotional distress, maladaptive coping, and fear-avoidance behaviors interact to maintain long-term impairment (2, 3). This perspective is reflected in research emphasizing that CLBP is frequently accompanied by psychological and social consequences (e.g., anxiety, depression, reduced wellbeing) and that successful management often requires interventions that address both physical function and pain-related cognitive–affective processes (4). Exercise-based approaches remain foundational in conservative CLBP management (5). Conventional exercise or physical therapy programs—often including strengthening, stretching, stabilization, and graded activity—are widely used to improve pain and disability. Within controlled trials, physical therapy interventions can yield meaningful functional gains, and they are commonly positioned as a reimbursable, evidence-based standard of care (6).

Yoga has emerged as a widely studied mind–body exercise option for CLBP (7). Yoga interventions typically integrate postures (asanas), breathing practices, relaxation, and sometimes meditation or lifestyle education (8). This combination is clinically relevant because CLBP outcomes are influenced by both physical factors (mobility, trunk strength, movement patterns) and psychological factors (stress reactivity, catastrophizing, pain acceptance, fear of movement) (9, 10). In a meta-analysis of randomized trials (RCTs) published earlier, yoga demonstrated medium-to-large post-treatment effects on both functional disability and pain, with smaller but significant follow-up effects (11). Importantly, that meta-analysis noted heterogeneity across yoga styles and dosing, yet post-treatment heterogeneity was low for key outcomes, suggesting that yoga’s short-term benefits might generalize across different program formats. Contemporary systematic reviews have expanded these findings, demonstrating yoga’s capacity to alleviate pain symptoms from immediate to mid-range follow-up intervals, while showing enhanced physical functioning benefits that persist from short-term through extended observation periods when contrasted with non-active control groups (12). A critical question, however, is not whether yoga is beneficial compared with minimal intervention or usual care, but whether yoga offers advantages over conventional exercise therapy.

At the same time, yoga may provide distinctive benefits on psychological outcomes that are central to CLBP persistence (13). Research examining pain-associated functional limitations and bodily flexibility revealed that participants in a 7-day immersive yoga retreat showed greater improvements in mobility and reduced impairment compared to those undergoing conventional physical therapy (14). Although such residential programs differ from typical outpatient care and may have limited generalizability, they support the hypothesis that yoga’s integrated components could influence affective and stress-related domains alongside physical measures.

Recent evidence further illustrates that when yoga is compared with PT and home exercise under similar follow-up timing, multiple outcomes may improve across all active interventions. In a three-arm randomized trial comparing yoga, PT, and home exercise in CLBP over 6 weeks, all groups improved in pain intensity and disability, pain sensitivity, central sensitization symptoms, anxiety, trunk muscle activation, and quality of life; no single approach was uniformly superior, although PT showed a greater improvement on disability and cortisol reduction occurred only in the PT arm (15). These findings suggest that several active movement-based approaches—whether yoga or exercise therapy—can generate multi-domain improvements, and that differences between programs may emerge for particular outcomes (e.g., disability change or biomarkers) rather than pain alone. Beyond symptom and function measures, cognitive appraisal and coping processes are increasingly recognized as modifiable targets linked to CLBP prognosis. In addition to conventional assessments of symptoms and physical function, cognitive evaluation and adaptive coping mechanisms are now acknowledged as influential factors that can be modified to influence CLBP outcomes. A supplementary examination of the Back to Health Trial revealed that yoga, PT, and educational interventions each contributed to enhanced pain self-efficacy by the 12-week mark, with statistically and clinically significant improvements particularly evident in the yoga and PT cohorts. Research findings indicated that participants in both yoga and physical therapy groups demonstrated the most substantial reduction in catastrophizing tendencies by the 12-week mark, with these positive effects persisting through the 52-week follow-up period (16). Notably, there were no statistically significant between-group differences in these cognitive outcomes, suggesting that structured education and active movement programs may share common mechanisms that improve self-efficacy and reduce catastrophizing (17). These observations hold clinical significance as enhanced cognitive processing was correlated with greater long-term progress in alleviating pain symptoms and functional limitations.

Based on existing research findings, yoga theoretically has the potential to improve spinal stiffness in patients with CLBP, alleviate pain, and provide psychological interventions. However, each randomized controlled trial (RCT) exhibits variations in the degree and aspects of improvement, primarily due to differences in the scales used, research designs, and intervention methods. These factors complicate our assessment of the effects of yoga compared to other exercise-based interventions on CLBP.

Consequently, Consequently, a comprehensive meta-analysis directly comparing yoga with conventional exercise therapies is warranted. This investigation would aim to determine the comparative effectiveness across key CLBP indicators—pain intensity, functional limitations, and mental wellbeing—while addressing variations in treatment protocols and evaluation criteria.

## Methods

### Protocol and reporting

This meta-analysis was conducted in accordance with the PRISMA statement and the Cochrane Handbook for Systematic Reviews of Interventions (18). This research was registered in PROSPERO (registration number: CRD420261283807).

### Literature search

An extensive search of multiple electronic databases was performed, covering publications from their earliest records through November 23, 2025. The investigation encompassed PubMed, Embase, Cochrane Library, Web of Science, and PEDro databases. The search methodology incorporated both standardized subject headings (where applicable) and keyword variations associated with yoga therapy and chronic low back pain. Key search parameters focused on “yoga” and its variations, along with terms describing chronic low back pain conditions. PubMed (MEDLINE) search strategy: ((“Yoga”[Mesh] OR yoga[Title/Abstract] OR yogic[Title/Abstract]) AND (“Low Back Pain” [Mesh] OR “chronic low back pain” [Title/Abstract] OR CLBP[Title/Abstract] OR “persistent low back pain” [Title/Abstract] OR “non-specific low back pain” [Title/Abstract])) AND (randomized controlled trial[Publication Type] OR controlled clinical trial[Publication Type] OR random*[Title/Abstract] OR trial[Title/Abstract] OR placebo[Title/Abstract]).

### Study selection

The complete set of search results was systematically organized using Endnote X9 software. Two researchers independently performed the screening and documented the excluded studies along with the reasons for exclusion. The research design specifically included parallel-group RCTs as the study type.

Eligible participants were adult individuals aged 18 years or older suffering from CLBP, characterized by persistent symptoms lasting a minimum of 12 weeks. Trials focusing on non-specific CLBP were eligible. Intervention: yoga as the primary intervention, with no restriction on yoga style (e.g., Hatha yoga, Iyengar yoga, integrated yoga). Comparator: exercise-based interventions (e.g., conventional therapeutic exercises, physiotherapy exercise programs, stabilization exercise programs). Comparators had to be active exercise interventions, not usual care alone. Outcomes: trials reporting at least one eligible continuous outcome in a form that allowed quantitative synthesis (mean and standard deviation (SD), change scores, or equivalent data) for one or more of these key areas: (1) severity of pain; (2) functional limitations related to spinal issues; (3) mental health or emotional wellbeing measures (when specified in the study design).

Exclusion criteria were: Non-randomized designs, quasi-experimental studies, observational studies, protocols, conference abstracts without full data, and narrative reviews. Trials in which yoga or the comparator exercise was combined with additional co-interventions (e.g., medication programs, manual therapy, acupuncture) that were not matched across groups, making the comparative effect of yoga versus exercise unclear. Studies without extractable outcome data (e.g., data only displayed in figures without numerical values and unavailable from authors). Duplicate publications using overlapping samples (in such cases, the most complete dataset was retained).

### Data extraction and management

The research team systematically collected and analyzed key information from the selected studies for quantitative evaluation. This comprehensive dataset encompassed multiple dimensions: researcher identities and study dates, geographical locations, participant age ranges across treatment and comparison cohorts, distinct therapeutic approaches implemented in each group, particular yoga styles employed in the experimental arm, frequency and length of intervention sessions, total treatment period, and assessment criteria. Two investigators independently performed this data collection procedure. Following information gathering, they collaboratively developed a structured data compilation framework, which subsequently underwent verification by an additional researcher. Through collective deliberation among all three team members, consensus was reached regarding the definitive dataset and the organization of the compiled information.

### Quality assessment

Two investigators separately performed bias risk evaluations utilizing Review Manager 5.4 software. Following comprehensive article review, they appraised potential biases in the selected research papers. The assessment focused on multiple domains: selection bias (including randomization methods and concealment procedures), performance bias (related to participant and staff blinding), detection bias (concerning outcome evaluator blinding), attrition bias (pertaining to missing data), and reporting bias (involving selective result disclosure). Each criterion was rated according to three categories: (1) minimal bias risk, (2) significant bias risk, or (3) indeterminate bias risk. Upon finishing individual evaluations, the researchers reconciled any discrepancies through deliberation and consultation with a third reviewer.

### Data analysis

The research employed Review Manager 5.4, a meta-analysis tool developed by the Cochrane Collaboration, to process all collected study data. Since the dataset exclusively comprised continuous variables, the analysis utilized standardized mean differences (SMD) for effect size estimation. Statistical significance was determined through z-tests with 95% confidence intervals (CIs).

To evaluate inter-group variability, the analysis incorporated both Cochran’s Q test and I^2^ statistics. In cases where the Q test yielded *P*-values exceeding 0.05 or I^2^ values remained below 50%, indicating minimal heterogeneity, a fixed-effects model was implemented. Conversely, when these thresholds were exceeded, a random-effects model was selected. The I^2^ metric served as the primary measure for quantifying study-to-study variation, with values surpassing 50% denoting significant heterogeneity.

For instances where I^2^ exceeded 50%, additional sensitivity analyses were performed to pinpoint potential causes of variability and offer interpretive insights. Throughout the analysis, statistical significance was established at *P*-values below 0.05.

## Results

### Search results

**FIGURE 1:**
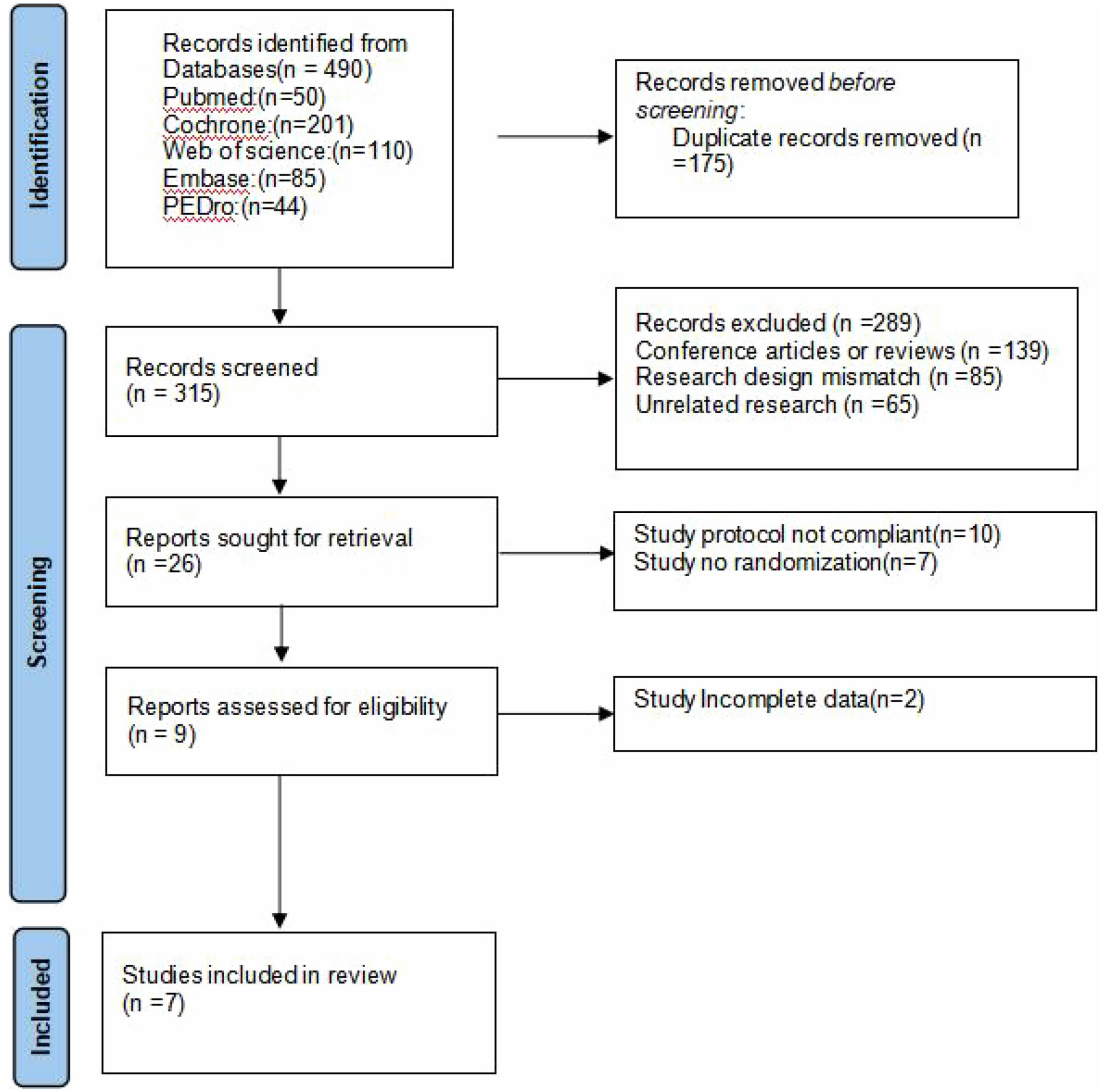
Literature search and screening flow diagram.

The systematic literature review process is illustrated in Figure 1. Our search strategy yielded 490 potentially relevant publications from five major medical research databases (PubMed: 50 records, Cochrane Library: 201 records, Web of Science: 110 records, Embase: 85 records, PEDro: 44 records). These references were systematically organized using EndNote X9 reference management software. Following the removal of 175 duplicate entries, 315 unique publications remained for preliminary evaluation. The screening procedure involved rigorous assessment of titles and abstracts based on PICO criteria, resulting in the exclusion of 139 review papers and conference proceedings, 85 non-randomized studies, and 65 publications deemed irrelevant to our research focus. Subsequently, we conducted full-text evaluations of 26 selected articles. During the detailed examination phase, 10 articles were eliminated for failing to meet methodological requirements, while 7 additional studies were excluded due to inadequate randomization procedures. The data extraction process led to the exclusion of 2 more studies with insufficient data. Ultimately, our meta-analysis incorporated 7 high-quality randomized controlled trials that satisfied all inclusion criteria.

### Study characteristics

Seven randomized controlled trials were included, comparing yoga interventions with exercise-based comparators for adults with CLBP. Five studies were conducted in India (14, 19–22), one in the United States (23), and one in Turkey (15), as detailed in Table 1. Study populations primarily consisted of middle-aged individuals, with average participant ages falling between 31 and 49 years across different trials. The number of subjects involved showed considerable variation, ranging from approximately 30–256 participants. Yoga styles included traditional yoga, Iyengar yoga, integrated yoga, and Hatha yoga, with marked variation in dose and duration, ranging from intensive short-term programs delivered daily for 1 week (80 min/session, 7 sessions/week) to outpatient-style protocols lasting up to 12 weeks (e.g., 75 min/session, once weekly). Comparator conditions were active exercise interventions (physical/physiotherapy exercise or conventional therapeutic exercise), and one trial used a home-exercise program, indicating variability in supervision and treatment contact (15). Outcomes were heterogeneous and spanned multiple domains: pain intensity (VAS or DVPRS), back-related disability/function (ODI or RDQ), psychological constructs (e.g., anxiety assessed by STAI; pain self-efficacy assessed by PSEQ), and quality of life (HRQOL or WHOQOL-BREF), which may contribute to clinical and methodological heterogeneity in pooled analyses. Comprehensive data regarding the selected clinical studies’ attributes are presented in Table 1.

**TABLE 1:** Basic characteristics of included citations.

Author, year	Country	E/C(N)	Age (year) (M ± SD)	Experimental group	Control group	Yoga type	Time/times/weeks of yoga	Outcome
Tekur,2008	India	40/40	E:49 ± 3.6 C:48 ± 4	Yoga	Physical exercises	Traditional yoga	480 min/7/w/1 w	ODI
Tekur,2012	India	40/40	E:49 ± 3.6 C:48 ± 4	Yoga	Physical exercises	Traditional yoga	480 min/7/w/1 w	STAI,VAS
Nambi,2014	India	30/30	E:44.26 ± 9.26 C:43.66 ± 8.82	Yoga	Physical exercise	Iyengar yoga	30 min/6/w/4 w	VAS,HRQOL
Nagaratna,2018	India	44/44	E:31.45 ± 3.47 C:32.75 ± 3.71	Yoga	Physical exercise	Integrated yoga	60 min/5/w/6 w	WHOQOL-BREF
Neyaz,2019	India	35/35	E:38 ± 12.22 C:33 ± 12.22	Yoga	Conventional therapeutic exercises	Hatha yoga	60 min/6/w/6 w	DVPRS,RDQ
Marshall,2022	USA	127/129	E:46.7 ± 10.2 C:46.0 ± 11.4	Yoga	Physical exercise	Hatha yoga	75 min/12/w/12 w	PSEQ
Oz,2024	Turkey	43/40	E:41 ± 8.90 C:41 ± 13.33	Yoga	Home exercise	Hatha yoga	60 min/3/w/6 w	VAS,ODI

E, Experimental group; C, Control group; M, Mean; SD, Standard Deviation; VAS, Visual Analog Scale; ODI, Oswestry Disability Index; STAI, State-Trait Anxiety Inventory; HRQOL, Health-Related Quality of Life; WHOQOL-BREF, World Health Organization Quality of Life Brief Version; DVPRS, Defense and Veterans Pain Rating Scale; RDQ, Roland-Morris Disability Questionnaire; PSEQ, Pain Self-Efficacy Questionnaire. Intervention Dosage (Session Duration/Frequency/Total Duration).

### Quality assessment

**FIGURE 2:**
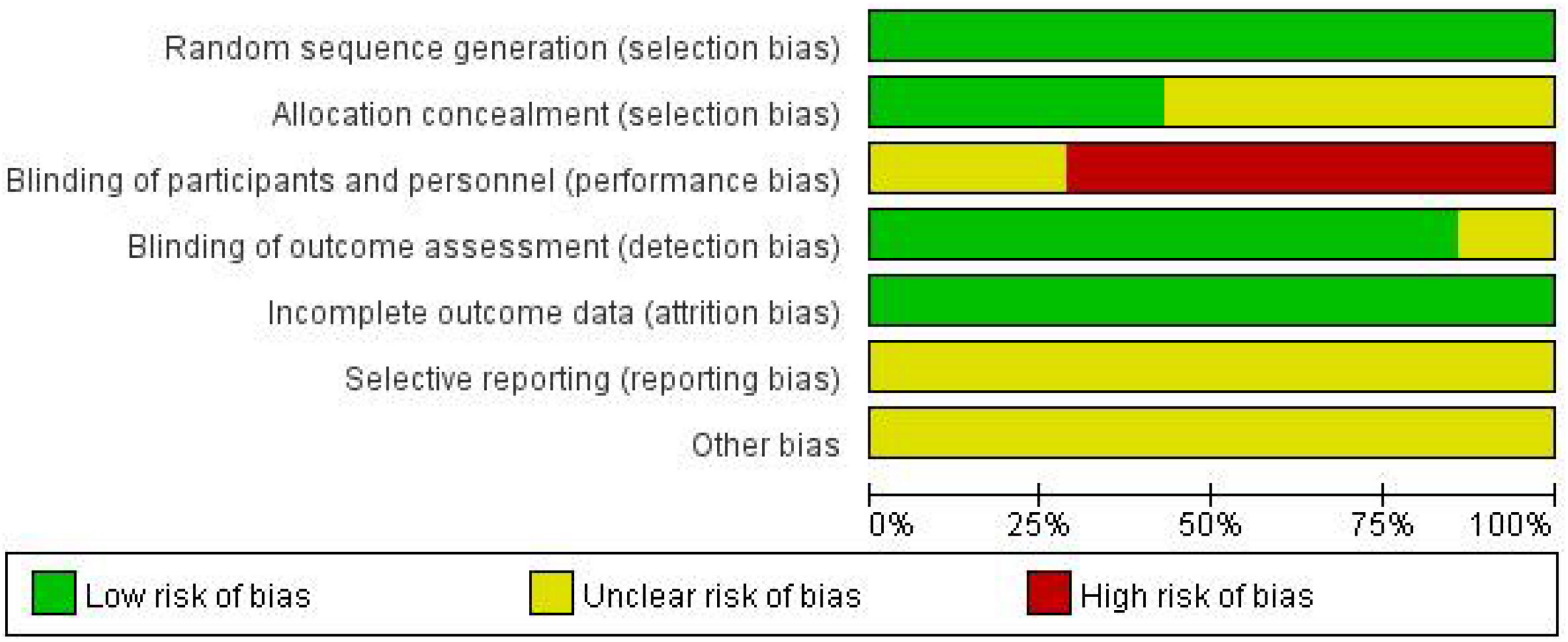
Risk of bias summary for included studies.

**FIGURE 3:**
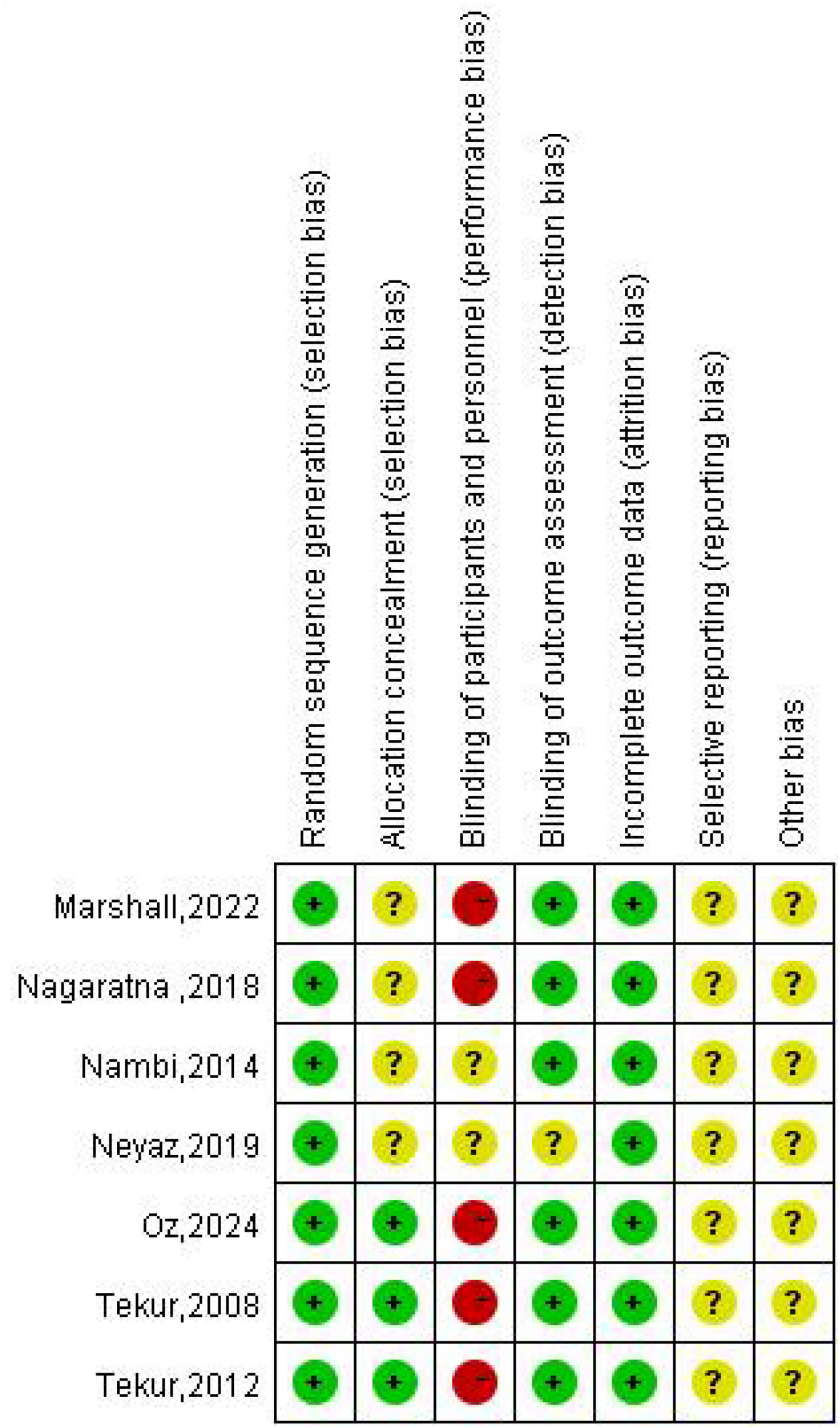
Judgments about each risk of bias item for all included studies.

The evaluation of potential bias was conducted utilizing the Cochrane risk assessment tool (Figures 2, 3). In summary, all seven included studies demonstrated minimal risk concerning the generation of random sequences, reflecting proper documentation of randomization methods. Allocation concealment was less consistently described: three studies were rated low risk (14, 15, 22), whereas the remaining trials were judged unclear due to insufficient methodological detail. Because yoga and exercise interventions are difficult to blind, blinding of participants and personnel was rated high risk in most studies, representing the primary limitation in internal validity. In contrast, blinding of outcome assessment was predominantly low risk, suggesting that detection bias may have been mitigated in several trials. Incomplete outcome data was generally rated low risk, with limited evidence of differential attrition between groups. However, selective reporting and other bias domains were frequently judged as unclear, largely reflecting limited protocol availability and incomplete reporting of prespecified outcomes or analytical decisions. Collectively, the evidence base shows acceptable methodological quality in randomization and attrition handling, but performance bias and reporting transparency remain key concerns when interpreting pooled estimates.

Interpreting these risk of bias findings, the predominant methodological limitation across the evidence base is the high risk of performance bias. Because it is virtually impossible to blind participants and instructors to active exercise or yoga interventions, self-reported subjective outcomes—such as pain intensity and emotional wellbeing—are inherently susceptible to participant expectation or placebo effects. This structural limitation is common in non-pharmacological trials but must be considered when evaluating the magnitude of the reported effect sizes.

### Yoga on pain

**FIGURE 4:**
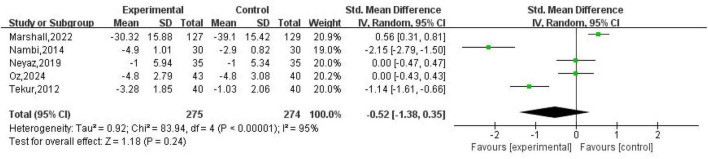
Forest plot of the effect of yoga on pain compared to control group.

Five studies (15, 19, 20, 22, 23) reported pain-related outcomes, involving 549 participants, as illustrated in Figure 4. Statistical analysis revealed no statistically meaningful difference in pain reduction between yoga practitioners and control groups (Standardized Mean Difference = –0.52; 95% Confidence Interval = -1.38 to 0.35; *P* = 0.24, with substantial heterogeneity indicated by *I*^2^ = 95%).

### Yoga on physical function

**FIGURE 5:**
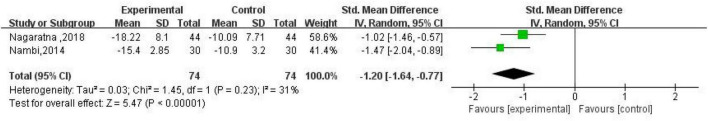
Forest plot of the effect of yoga on physical function in patients with CLBP.

Two studies (19, 20) assessed changes in physical function in 148 CLBP patients, presented in Figure 5. The findings demonstrated statistically significant functional enhancement in the yoga intervention group compared to controls, with minimal heterogeneity observed (Standardized Mean Difference = -1.2; 95% Confidence Interval = -1.64 to –0.77; *P* < 0.00001; *I*^2^ = 31%).

### Yoga on disability

**FIGURE 6:**
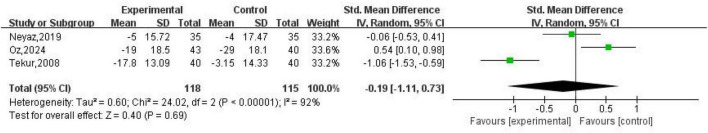
Forest plot of the effect of yoga on disability in patients with CLBP.

Three research investigations (14, 15, 20) involving 233 individuals with CLBP examined alterations in physical capabilities following yoga intervention, as illustrated in Figure 6. The analysis revealed no statistically meaningful variation in physical functionality between the yoga and control cohorts (Standardized Mean Difference = –0.19; 95% Confidence Interval: -1.11 to 0.73; *p* = 0.69; Heterogeneity Index = 92%).

### Yoga on emotion

**FIGURE 7:**
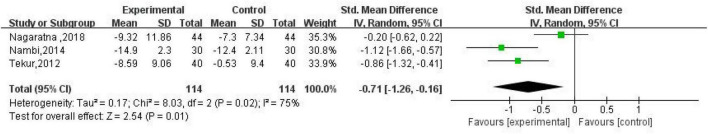
Forest plot of the effect of yoga on emotional wellbeing in patients with CLBP.

A series of three clinical trials (19, 20, 22) assessed the psychological status of 228 participants, with findings presented in Figure 7. The meta-analysis demonstrated that yoga practitioners exhibited substantially better emotional outcomes relative to the control group (SMD = –0.71; 95% CI: -1.26 to –0.16; *p* = 0.01; *I*^2^ = 75%). Sensitivity analysis conducted after removing one particular study (20) maintained the significant beneficial impact of yoga on emotional health in CLBP patients (SMD = –0.97; 95% CI: –1.32 to –0.62; *P* < 0.00001; *I*^2^ = 0%).

## Discussion

This comprehensive review and meta-analysis examined data from seven randomized controlled trials that evaluated the effectiveness of yoga versus exercise-based interventions for CLBP. Overall, yoga was not superior to exercise controls for pain, showed a significant benefit for physical function in a small evidence set, produced no clear advantage for disability (“disability”), and was associated with a moderate improvement in emotional outcomes that became more consistent after removal of one influential trial. Taken together, these findings support the view that yoga is a credible active intervention for CLBP, but its comparative effects versus other forms of exercise appear outcome-dependent and are strongly shaped by heterogeneity in interventions, comparators, and measurement.

### Yoga on pain

The pooled estimate for pain did not reach statistical significance and exhibited extreme heterogeneity (*I*^2^ = 95%). This pattern is consistent with prior comparative syntheses suggesting that yoga tends to outperform non-exercise controls but often performs similarly to structured exercise therapy when directly compared (24). Large heterogeneity is also compatible with earlier meta-analytic work indicating variability in yoga trial effects across populations and program formats (25). Clinically, pain intensity in CLBP is influenced by baseline severity, psychological distress, and contextual factors; therefore, mixing short, intensive residential protocols with longer outpatient programs may lead to genuinely different pain trajectories rather than sampling noise alone (26). Comparator intensity may further amplify heterogeneity: supervised physiotherapy exercise is likely to deliver larger benefits than minimally monitored home exercise (27), reducing the apparent incremental benefit of yoga in some trials while enlarging it in others. Additionally, pain was measured using different instruments (e.g., VAS vs. DVPRS), and although SMD pooling addresses scale differences, it cannot remove construct-level differences in how pain is captured across contexts.

From a practical perspective, the non-significant pooled pain effect does not imply yoga is ineffective; rather, it suggests that when compared against active exercise controls, yoga’s incremental advantage for pain may be small or inconsistent. These findings are consistent with comparative clinical studies demonstrating yoga’s non-inferiority to physical therapy in addressing both pain and functional improvement, as well as randomized controlled trials showing comparable pain relief between traditional Hatha yoga and standard exercise therapies (28, 29). Therefore, improvements in pain outcomes may be attributed more to “participation in a structured movement program” rather than to any specific type of exercise.

### Yoga on function and disability

This analysis demonstrates the varied effects of yoga on mobility limitations in individuals with CLBP. The significant improvement in physical function observed in the yoga group suggests that its structured approach, incorporating specific postures and breathing techniques, effectively enhances physical capability (30). This improvement may be linked to the targeted engagement of core muscles, which promotes better spinal support and stability. As a result, patients experience greater flexibility and strength, both critical factors in effectively managing CLBP (31).

However, the analysis of functional impairment revealed no statistically significant differences between participants practicing yoga and those in the control group. This raises questions about yoga’s ability to address the multifaceted nature of disability. While physical function may improve, yoga alone might not be sufficient to mitigate the psychological and social dimensions of disability that often accompany chronic pain conditions. Patients frequently experience anxiety, depression, and fear related to their pain, which can perpetuate feelings of disability (32). Therefore, despite the physical benefits, these psychological factors may require additional interventions for comprehensive management.

The high heterogeneity observed in the disability outcomes underscores the variability in individual responses to yoga. Factors such as personal coping strategies, mental health status, and social support could influence how patients perceive their disability. In this context, the group-based nature of yoga practice offers potential advantages by fostering a supportive community (33). The emotional and motivational support from peers may enhance adherence to the practice, contributing to improved physical outcomes. Nonetheless, the lack of a significant impact on disability highlights the need to integrate psychological support into yoga programs (34). Incorporating elements such as mindfulness, cognitive-behavioral approaches, or stress management techniques could create a more holistic treatment framework.

Further investigation into different styles of yoga and their specific effects on both physical function and perceived disability is warranted. Understanding how variations in yoga practices influence patient outcomes will be crucial in refining treatment protocols (35). Exploring the optimal duration and frequency of yoga sessions may also provide insights for maximizing benefits for individuals with CLBP. By addressing both physical and psychological dimensions through tailored interventions, practitioners can aim to achieve more comprehensive improvements in overall patient wellbeing.

### Yoga on emotional

The research findings indicate that yoga practice yields beneficial effects on the psychological state of individuals suffering from chronic low back pain. Analysis of three clinical trials involving 228 participants showed markedly better emotional health outcomes among those engaged in yoga practice compared to non-participating counterparts. These observations highlight yoga’s therapeutic value in promoting mental wellness. The observed variations in results could be attributed to differences in yoga techniques employed, session lengths, and individual patient factors, each potentially influencing psychological benefits.

Even after excluding one study (20) from the analysis, the remaining results continued to support yoga’s effectiveness in fostering emotional wellbeing, with an even greater standardized mean difference (SMD) of –0.97. This robust finding reinforces the notion that yoga serves as a viable therapeutic option for patients dealing with the emotional burdens associated with CLBP. The absence of heterogeneity (*I*^2^ = 0%) in this refined analysis highlights the consistency of positive effects across the remaining studies, suggesting a strong and uniform benefit of yoga practice on emotional health.

Yoga’s multifaceted approach, which combines physical movement, breath control, and mindfulness, likely contributes to these emotional benefits. The practice encourages a state of relaxation and mindfulness, allowing individuals to manage stress and anxiety more effectively (36, 37). As patients engage in yoga, they cultivate greater body awareness and emotional regulation skills, which can lead to improved mood and overall emotional resilience (38).

Enhancing emotional wellbeing is crucial for patients with CLBP, as emotional distress can exacerbate pain perception and hinder recovery (39). Incorporating yoga interventions within therapeutic regimens allows healthcare professionals to provide comprehensive care targeting both physiological and psychological aspects of chronic pain. Such integrated treatment strategies can enhance patient autonomy, encouraging active participation in the recovery journey.

### Limitations

The current research has certain constraints that warrant consideration. Primarily, in pursuit of high-quality evidence, it included only RCTs, which resulted in a relatively small number of studies and may introduce some bias in the findings. Additionally, the variety of yoga styles, as well as differences in treatment duration and frequency, could lead to varying therapeutic effects. Formal subgroup analyses could not be performed due to the limited number of trials per outcome and inconsistent reporting of subgroup-defining variables. Furthermore, because of the small number of included trials for key outcomes (e.g., only five for pain and three for disability), robust sensitivity analyses (such as systematic leave-one-out procedures) or meta-regressions could not be meaningfully performed to statistically pinpoint the exact sources of the substantial heterogeneity (*I*^2^ > 90%). Consequently, the high heterogeneity remains largely unexplained, which limits the reliability of the pooled estimates. A key limitation of this review is that the meta-analysis for physical function was informed by only two trials. This restricted evidence base reduces statistical power, limits exploration of heterogeneity, and increases the likelihood that the pooled effect is unstable or overly influenced by study-specific features (e.g., intervention dose, supervision intensity, or baseline severity). Consequently, any apparent functional advantage of yoga over exercise should be interpreted cautiously and should not be regarded as definitive until confirmed by additional adequately powered RCTs using standardized functional outcome measures and complete reporting.

## Conclusion

In this meta-analysis, yoga showed favorable outcomes in physical function and emotional wellbeing when compared to exercise-based controls, though its effects on pain intensity and disability were comparable to exercise. It is important to note that these results are derived from a restricted set of trials and are characterized by substantial heterogeneity. Consequently, these findings represent the statistical evidence captured in this specific study and should be interpreted as a basis for future large-scale research rather than a generalized assertion of yoga’s superiority.

## Data Availability

The original contributions presented in the study are included in the article/supplementary material, further inquiries can be directed to the corresponding author.
